# Supplementary material for: Synthetic Routes to 2-aryl-1H-pyrrolo[2,3-b]pyridin-4-amines: Cross-Coupling and Challenges in SEM-Deprotection
Source: Molecules. 2024 Oct 7;29(19):4743. doi: 10.3390/molecules29194743 (PMC11478076; doi:10.3390/molecules29194743)
Supplement: Supplementary file 1 [file molecules-29-04743-s001.zip › molecules-3228810-supplementary.pdf]

Supplementary Material

Synthetic routes to 2-aryl-1*H*-pyrrolo[2,3-*b*]pyridin-4-amines: cross-coupling, and challenges in SEM-deprotection

Srinivas Reddy Merugu [1], Sigrid Selmer Olsen [1], Camilla Johansen Kaada [1], Eirik Sundby [2], and Bård Helge Hoff [1,*]

[1] Department of Chemistry, Norwegian University of Science and Technology (NTNU), N-7491 Trondheim, Norway, e-mail: bard.h.hoff@ntnu.no.

[2] Department of Materials Science and Engineering, Norwegian University of Science and Technology (NTNU), N-7491 Trondheim, Norway, e-mail: eirik.sundby@ntnu.no.

*Correspondence: bard.helge.hoff@ntnu.no

Contents

1. Description for synthesis of building blocks 2
2. Experimental procedures for building blocks and side-products 3
3. Biochemical assays 10
4. NMR spectra 12
5. References 28

1. Description for synthesis of building blocks

To prepare the core 7-azaindole building block **4** we oxidized 7-azaindol using *m*-chloroperbenzoic acid (m-CPBA) to form the corresponding *N*-oxide followed by a chlorinated as shown in Scheme S1. This gave a mixture of two isomeric as previously reported [1]. At best we achieved a ratio of 25/1 in favour of the target compound **4**. However, due to difficulties in removing the isomer, resulting in only 50% yield, it was found more convenient to proceed with purchased 4-chloro-1*H*-pyrrolo[2,3-*b*]pyridine (**4**).

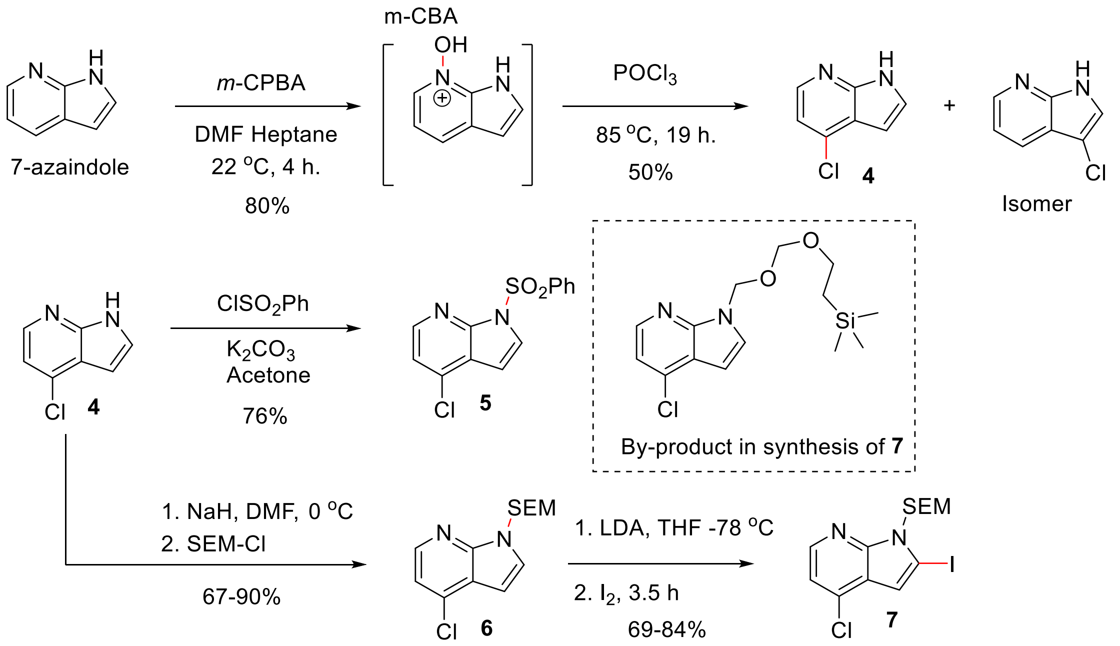

Scheme S1. Synthesis of the key building blocks.

To enable iodine insertion at C-2 a *N*-1 directing group is needed. Two groups were considered, benzene sulfonyl and 2-(trimethylsilyl)ethoxymethyl (SEM). 4-Chloro-7-azaindol (**4**) was protected as the benzene sulfonyl derivative **5** by the procedure of Layek *et al.*[2]. In synthesis of the corresponding SEM derivative **6**, which is also a known compound[3], reactions up to 9 grams scale were performed. Dry conditions are essential for attaining good conversion and yield. We also isolated small amounts of the corresponding 2-(trimethylsilyl)ethoxy)-methoxy)methyl derivative. An impurity in the starting material was observed, which is the likely case of this side-product. However, this impurity might also be caused by reaction with DMF [4], forming a hydroxymethyl derivative undergoing reaction with SEM chloride. Directed lithiation at -78 °C followed by quenching with iodine gave the key building block **7**

in 69-84% yield in up to 9 g scale. Strictly dry conditions are also here essential to obtained high conversion and yield. A similar iodination has previously been done on the corresponding methoxymethyl protected analogue [5].

2. Experimental procedures for building blocks and side-products

4-Chloro-1*H*-pyrrolo[2,3-*b*]pyridine (4)[6]

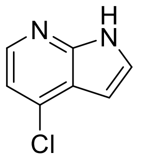

7-Azaindole (2.01 g, 17.0 mmol) was dissolved in DME/heptane (1:2, 21.5 mL). 3-Chloroperbenzoic acid (3.35 g, 21.4 mmol) was added portion-wise at 8-26 °C. The slurry was stirred for 3 h at rt (22 °C) under N_2 atmosphere before more 3-chloroperbenzoic acid (1.20 g, 7.66 mmol) and DME/*n*-heptane (1:2, 10 mL) were added. The mixture was stirred for another 45 min, before the mixture was filtered. The solid isolated was washed with DME/heptane (1:2, 50 mL) and concentrated in vacuo to give the 7-hydroxy-1*H*-pyrrolo[2,3-*b*]pyridinium 3-chlorobenzoate as an off-white solid, 3.94 g (13.5 mmol, 80%), *mp.* 139 - 141 °C (lit. [6] 141 – 143 °C). To 7-hydroxy-1*H*-pyrrolo[2,3-*b*]pyridinium 3-chlorobenzoate (2.01 g, 6.90 mmol) was added $POCl_3$ (4.75 mL, 50.9 mmol) at room temperature. The mixture was heated slowly to 85 °C and stirred for 19 h, before the mixture was cooled to 0 °C. The mixture was added sat. aq. $NaHCO_3$ (14 mL). The solvent was removed in vacuo before the mixture adjusted to pH 13 with NaOH solution (5 M). The mixture was filtered and the solid obtained was re-slurried with water and filtered again. The crude material was then dried. The product was purified by silica-gel column chromatography (CH_2Cl_2/MeOH, 97:3, R_f = 0.14) to give an off-white solid, 0.53 g (3.44 mmol, 50%), mp. 171.0 - 173 °C (Lit.[6] 176 – 177 °C). ^{1}H NMR (400 MHz, DMSO-*d*$_6$) δ 12.03 (s, br, 1H), 8.17 (d, J = 5.2 Hz, 1H), 7.60-7.58 (ap.d, 1H), 7.19 (d, J = 5.2 Hz, 1H), 6.51-6.50 (ap.d, 1H). The shifts correspond with previously reported data [7].

4-Chloro-1-(phenylsulfonyl)-1*H*-pyrrolo[2,3-*b*]pyridine (5)

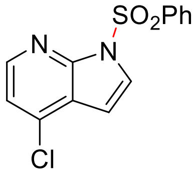

A mixture of 4-chloro-7-azaindole (**4**) (96 mg, 0.63 mmol) and K_2CO_3 (229 mg, 1.65 mmol) was dissolved in acetone (3.1 mL) and cooled to 0 °C. The mixture was stirred for 10 minutes before benzenesulfonyl chloride (0.13 mL, 0.98 mmol) was added dropwise over 5 min. The reaction mixture stirred at 0 °C for 10 more min. before it was heated at 58 °C for 18 h. The mixture was the filtered at 30 °C to remove solids, and the filtrate was concentrated in vacuo, before CH_2Cl_2 (15 mL) and water (10 mL) was added. The layers were separated, and the aqueous layer was extracted with more CH_2Cl_2 (3 × 15 mL). The combined organic phases were washed with brine (20 mL) and dried over anhydrous Na_2SO_4, filtered and concentrated in vacuo to the product as a beige solid, 141 mg (0.482 mmol, 76%); mp. 120-121 °C (lit.[2] 120 °C); ^{1}H NMR (400 MHz, DMSO-*d*$_6$): 8.35 (d, J = 5.3 Hz, 1H), 8.13-8.11 (ap.d, 2H), 8.06 (d, J = 4.1 Hz, 1H), 7.76-7.72 (ap.t, 1H), 7.64 (t, J = 7.7 Hz, 2H), 7.48 (d, J = 5.3 Hz, 1H), 6.90 (d, J = 4.1 Hz, 1H). The shifts correspond with previously reported data [2].

4-Chloro-1-((2-(trimethylsilyl)ethoxy)methyl)-1*H*-pyrrolo[2,3-*b*]pyridine (6) [8]

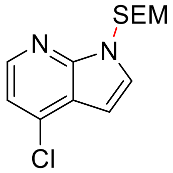

4-Chloro-1*H*-pyrrolo[2,3-*b*]pyridine (**4**) (5.43 g, 35.6 mmol) was dissolved in dry DMF (47 mL). Then NaH (1.24 g, 51.8 mmol) was added to the reaction flask under an N_2 atmosphere at 0 ºC. After stirring for 30 min at 0 ºC, 2-(trimethylsilyl)ethoxymethyl chloride (7.1 mL, 40.7 mmol) was added dropwise over 10 min. The reaction mixture was stirred at 0 ºC for 3 h and 45 min, before being allowed to warm rt. The mixture then was quenched with sat. aq. NH_4Cl (100 mL) and extracted with EtOAc (3×100 mL). The combined organic phases were washed with brine (100 mL), dried over anhydrous Na_2SO_4, filtered and concentrated in vacuo. The product was purified by silica gel column chromatography (*n*-pentane/EtOAc, 96:4, R_f = 0.38) to give compound **1** as a light-yellow oil, 9.11 g (32.2 mmol, 90%); ^{1}H NMR (400 MHz, DMSO-*d*$_6$) δ 8.25 (d, *J* = 5.2 Hz, 1H), 7.78 (d, *J* = 3.6 Hz, 1H), 7.28 (d, *J* = 5.2 Hz, 1H), 6.60 (d, *J* = 3.6 Hz, 1H), 5.64 (s, 2H), 3.51 (t, *J* = 7.9 Hz, 2H), 0.81 (t, *J* = 7.9 Hz, 2H), -0.11 (s, 9H). The reported shifts correspond well with previously reported data [8].

4-Chloro-1-(((2-(trimethylsilyl)ethoxy)methoxy)methyl)-1*H*-pyrrolo[2,3-*b*]pyridine

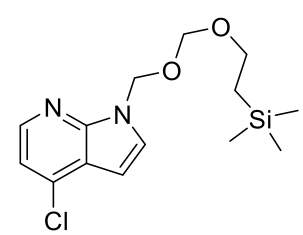

Following the procedure described in synthesis of **6**, this compound was isolated by column chromatography (*n*-pentane/EtOAc, 94:6, R_f = 0.17) as a yellow oil, 133 mg (0.425 mmol, 1%); ^{1}H NMR (400 MHz, $CDCl_3$) δ 8.25 (d, J = 5.2 Hz, 1H), 7.38 (d, J = 3.6 Hz, 1H), 7.13 (d, J = 5.2 Hz, 1H), 6.61 (d, J = 3.6 Hz, 1H), 5.77 (s, 2H), 4.74 (s, 2H), 3.55 (t, J = 8.6 Hz, 2H), 0.87 (t, J = 8.6 Hz, 2H), -0.01 (s, 9H); ^{13}C NMR (100 MHz, $CDCl_3$) δ 148.6, 143.8, 136.1, 128.8, 120.2, 116.7, 99.6, 92.9, 70.8, 65.7, 18.0, -1.5 (3C); HRMS (APCI/ASAP, m/z): found 313.1142, calcd. for $C_{14}H_{22}N_2O_2ClSi$, $[M+H]^+$, 313.1139.

4-Chloro-2-iodo-1-((2-(trimethylsilyl)ethoxy)methyl)-1H-pyrrolo[2,3-b]pyridine (7)

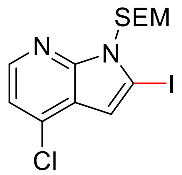

4-Chloro-1-((2-(trimethylsilyl)ethoxy)methyl)-1*H*-pyrrolo-[2,3-*b*]pyridine (**6**) (7.87 g, 27.8 mmol) was dissolved in dry THF (120 mL) under an N_2 atmosphere before cooling to -78 °C. Lithium diisopropylamide (2M in THF/*n*-heptane/ethylbenzene) (20 mL, 40 mmol) was added dropwise over a period of 45 min using a syringe pump. The reaction mixture was stirred at -78 °C for 1 h. Iodine (9.11 g, 35.9 mmol) dissolved in dry THF (40 mL) was then added dropwise over 45 min using a syringe pump. The mixture was then stirred for 2 h at -78 °C before being warmed to rt. The reaction was then quenched with sat. aq. NH_4Cl (2 mL). The solvent was removed in vacuo before addition of CH_2Cl_2 (200 mL) and water (200 mL). The two layers were separated, and the aqueous phase was extracted with more CH_2Cl_2 (6 × 50 mL). The combined organic phases were washed with brine (100 mL), dried over anhydrous Na_2SO_4, filtered and concentrated in vacuo. The crude product was dissolved in CH_2Cl_2 (100 mL), and added aq. $Na_2S_2O_3$ (10%, 50 mL). The water phase was extracted with CH_2Cl_2 (3 × 50 mL). The combined organic phases were dried over anhydrous Na_2SO_4, filtered and concentrated in vacuo. The product was purified by silica-gel column chromatography (*n*-pentane/EtOAc, 97.5:2.5, R_I= 0.37) to give compound **7** as a beige powder, 10.2 g (24.5 mmol, 69%); *mp*. 47.5-48.5 °C; ^{1}H NMR (400 MHz, DMSO-d_6) δ 8.19 (d, *J* = 5.2 Hz, 1H), 7.27 (d, *J* = 5.2 Hz, 1H), 6.99 (s, 1 H), 5.63 (s, 1H), 3.52 (t, *J* = 8.0 Hz, 2H), 0.81 (t, *J* = 8.0 Hz, 2H), -0.12 (s, 9H); ^{13}C NMR (100 MHz, DMSO-d_6) δ 149.4, 143.6, 132.9, 120.5, 116.9, 109.0, 89.8, 73.3, 65.8, 17.1, -1.3 (3C). IR (cm^{-1}, neat): 3129 (w), 2950 (w), 2908 (w), 1589 (m), 1556 (s), 1455 (s), 1381 (s), 1365 (s), 1074 (s, br), 850 (s, br), 755 (s), 740 (s): HRMS (APCI/ASAP, *m/z)*: found

409.0000, calcd. for $C_{13}H_{19}N_2OSiClI$, $[M+H]^+$, 409.0000. Spectroscopic data have not been reported in the literature.

***N*-Benzyl-4-chloro-*N*-methyl-1-((2-(trimethylsilyl)ethoxy)methyl)-1*H*-pyrrolo[2,3-*b*]-pyridin-2-amine (8)**

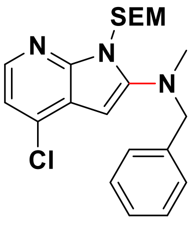

Following the Buchwald amination of compound **7** this compound was isolated in semi-pure form by column chromatography (*n*-pentane/EtOAc, 95:5, $R_f = 0.26$) as a yellow oil, 1.2 mg (0.0030 mmol, 1%). ^{1}H NMR (400 MHz, $CDCl_3$): 8.05 (d, J = 5.3 Hz, 1H), 7.35-7.28 (m, 5H), 7.04 (d, J = 5.3 Hz, 1H), 5.87 (s, 1H), 5.61 (s, 2H), 4.42 (s, 2H), 3.76 (t, J = 8.3 Hz, 2H), 2.80 (s, 3H), 0.94-0.88 (m, 2H), -0.06 (s, 9H); HRMS (APCI/ASAP, m/z): found 402.1761, calcd. for $C_{21}H_{29}N_3OSiCl$, $[M+H]^+$, 402.1768.

***N*-Benzyl-N-methyl-1*H*-pyrrolo[2,3-*b*]pyridin-4-amine (9)[9]**

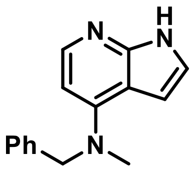

To a mixture of 4-chloro-7-azaindole (**4**) (101 mg, 0.663 mmol), *N*-benzyl-methylamine (0.13 mL, 1.01 mmol), RuPhos (5.2 mg, 0.011 mmol) and RuPhos Pd G2 (6.8 mg, 0.009 mmol), LiHMDS (1M in THF, 1.6 mL) were added under an N_2 atmosphere. The reaction was stirred at 65 °C for 5 h, before adding HCl (1M, 1.3 mL) and diluted with EtOAc (5 mL). The mixture was poured into sat.aq. $NaHCO_3$, and extracted with EtOAc (3 × 15 mL). The combined organic phases were washed with brine (20 mL) and dried over anhydrous Na_2SO_4, filtered and concentrated in vacuo. The product was purified by silica-gel column chromatography (CH_2Cl_2 containing 3% MeOH) to give an orange solid, 52 mg (22.0 mmol, 33%); mp. 222 – 223 °C, (lit. [9] 168 °C); ^{1}H NMR (400 MHz, $CDCl_3$) δ 9.65 (s, br, 1H), 8.01 (d, J = 5.8 Hz, 1H), 7.38-7.28 (m, 5H), 7.04 (d, J = 3.7 Hz, 1H), 6.48 (d, J = 3.7 Hz, 1H), 6.29 (d, J = 5.8 Hz, 1H), 4.83 (s, 2H), 3.21 (s, 3H); ^{13}C NMR (100 MHz, DMSO-*d₆*) δ 151.1, 149.9, 144.0, 138.2, 128.8 (2C), 127.2, 126.7 (2C), 120.4, 107.8, 101.5, 99.6, 57.1, 39.2. The shifts do not correspond with previously reported data [9]; IR (neat, cm $^{-1}$): 3075 (w, br), 2779 (w, br),1592 (m), 1576 (s), 1512 (m), 1444 (m), 1403 (m), 1325 (m), 1200 (m), 1044 (m), 925 (m), 842 (s), 725 (s); HRMS (APCI/ASAP, m/z): found 238.1342, calcd. for $C_{15}H_{16}N_3$, $[M+H]^+$, 238.1344.

N-Benzyl-N-methyl-1-((2-(trimethylsilyl)ethoxy)methyl)-1H-pyrrolo[2,3-b]pyridin-4-amine (10)

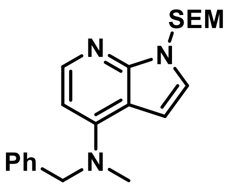

A mixture of 4-chloro-1-((2-(trimethylsilyl)ethoxy)methyl)-1*H*-pyrrolo[2,3-*b*]pyridine (**7**) (966 mg, 3.42 mmol), *N*-methyl-1-phenylmethylamine (0.6 mL, 4.6 mmol), NaO*t*-Bu (1.24 g, 12.9 mmol), RuPhos (83 mg, 0.18 mmol) and $Pd(OAc)_2$ (41.0 mg, 0.18 mmol) was added degassed *t*-BuOH (17 mL), under an N_2 atmosphere. The reaction mixture was stirred at 85 °C for 30 min before allowing to cool to room temperature. The solvent was removed in vacuo. Then CH_2Cl_2 (50 mL) and water (50 mL) were added to the flask and the layers were separated. The water phase was adjusted to pH 7 with *sat. aq*. NH_4Cl, and extracted with CH_2Cl_2 (3 × 50 mL). The combined organic phases were washed with brine (50 mL), dried over anhydrous Na_2SO_4, filtered and concentrated in vacuo. The product was purified by silica-gel column chromatography (*n*-pentane/EtOAc, 4:1, R_f =0.26) to give a light-yellow oil, 840 mg (2.29 mmol, 67%); ^1H NMR (400 MHz, DMSO-d_6) δ 7.88 (d, J = 5.7 Hz, 1H), 7.34-7.31 (m, 2H), 7.26-7.23 (m, 4H), 6.49 (d, J = 3.8 Hz, 1H), 6.28 (d, J = 5.7 Hz, 1H), 5.52 (s, 2H), 4.81 (s, 2H), 3.49 (t, J = 8.1 Hz, 2H), 3.19 (s, 3H), 0.81 (t, J = 8.1 Hz, 2H), -0.09 (s, 9H); ^{13}C NMR (100 MHz, DMSO-d_6) δ 149.7, 149.4, 143.9, 138.4, 128.5 (2C), 126.9, 126.5 (2C), 124.3, 107.3, 101.1, 99.8, 72.3, 65.1, 56.1, 39.6, 17.2, -1.4 (3C); IR (cm^{-1}, neat): 2950 (w), 2893 (w, br), 1703 (w), 1574 (s), 1503 (m), 1373 (m), 1246 (s), 1072 (s, br), 832 (s), 696 (s). HRMS (APCI/ASAP, *m/z*): found 368.2157, calcd. for $C_{21}H_{30}N_3OSi$, $[M+H]^+$, 368.2158.

1-((2-(Trimethylsilyl)ethoxy)methyl)-1H-pyrrolo[2,3-b]pyridine (11)

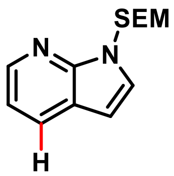

Following the procedure described for preparation of **10**, 1-((2-(trimethylsilyl)ethoxy)methyl)-1*H*-pyrrolo[2,3-b]-pyridine (**11**) was isolated by silica-gel column chromatography (*n*-pentane/EtOAc, 4:1, R_f = 0.75) as a yellow oil, 64 mg (0.26 mmol, 8%); ^1H NMR (400 MHz, $CDCl_3$) δ 8.33 (dd, J = 4.7, 1.5 Hz, 1H), 7.89 (dd, J = 7.8, 1.5 Hz, 1H), 7.34 (d, J = 3.6 Hz, 1H), 7.07 (dd, J = 7.8, 4.7 Hz, 1H), 6.50 (d, J = 3.6 Hz, 1H), 5.68 (s, 2H), 3.54 (m, 2H), 0.90 (t, J = 8.2 Hz, 2H), -0.08 (s, 9H). The shifts correspond with previously reported data [10]; HRMS (APCI/ASAP, m/z): found 249.1426, calcd. for $C_{13}H_{21}N_2OSi$, $[M+H]^+$, 249.1423.

***N*-Benzyl-2-iodo-*N*-methyl-1-((2-(trimethylsilyl)ethoxy)methyl)-1*H*-pyrrolo[2,3-*b*]pyridin-4-amine (12)**

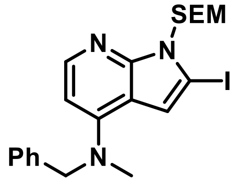

N-Benzyl-*N*-methyl-1-((2-(trimethylsilyl)-ethoxy)-methyl)-1*H*-pyrrolo[2,3-*b*]pyridin-4-amine (**10**) (746 mg, 2.03 mmol) was dissolved in dry THF (12.3 mL) under an N_2 atmosphere before cooling to -78 °C. LDA (2M in THF/*n*-heptane/ethylbenzene) (1.58 mL, 3.16 mmol) was added dropwise over 30 min using a syringe pump. The reaction mixture was stirred for 1 h before adding iodine (692 mg, 2.73 mmol) dissolved in dry THF (3.7 mL) dropwise over 30 min again using a syringe pump. The mixture was stirred for 3.5 h before being allowed to warm to room temperature. The pH was adjusted to pH 6 with HCl (1M) and sat. aq. $NaHCO_3$. The solvent was removed in vacuo before the mixture was added CH_2Cl_2 (30 mL) and water (20 mL). The layers were separated, and the aqueous layer was extracted with CH_2Cl_2 (3 × 30 mL). The combined organic phases were washed with brine (10 mL) and dried over anhydrous Na_2SO_4, filtered and concentrated in vacuo. The product was purified by silica-gel column chromatography (*n*-pentane/EtOAc, 5:1, R_f = 0.31) to give a yellow oil, 58 mg (0.12 mmol, 6%); ^{1}H NMR (400 MHz, $CDCl_3$) δ 7.96 (d, J = 5.7 Hz, 1H), 7.37 - 7.25 (m, 5H), 6.79 (s, 1H), 6.26 (d, J = 5.7 Hz, 1H), 5.66 (s, 2H), 4.74 (s, 2H), 3.60 (t, J = 8.2 Hz, 2H), 3.16 (s, 3H), 0.93 (t, J = 8.2 Hz, 2H), -0.06 (s, 9H); ^{13}C NMR (100 MHz, $CDCl_3$) δ 151.1, 149.3, 144.4, 137.7, 128.8 (2C), 127.3, 126.7 (2C), 111.9, 110.2, 100.6, 77.7, 73.3, 66.1, 57.1, 39.2, 17.8, -1.4 (3C); IR (neat, cm $^{-1}$): 2950 (w), 2892 (w, br), 1574 (s), 1451 (m), 1245 (m), 1072 (s), 832 (s), 694 (m); HRMS (APCI/ASAP, m/z): found 494.1125, calcd. for $C_{21}H_{29}N_3OSiI$, $[M+H]^+$, 494.1125.

4-(4-Chloro-1-((2-(trimethylsilyl)ethoxy)methyl)-1*H*-pyrrolo[2,3-*b*]pyridin-2-yl)benzyl formate (14f)

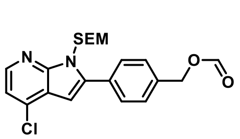

Compound **14f** was isolated as a side-product in the reaction to prepare **14e**. Purification by silica-gel column chromatography (*n*-pentane/EtOAc, 96:4, R_f = 0.13) gave 25 mg (0.060 mmol, 2%) of a clear oil;^{1}H NMR (400 MHz, DMSO-*d*$_6$) δ 8.37 (s, 1H), 8.28 (d, *J* = 5.2 Hz, 1H), 7.83 (d, *J* = 8.3 Hz, 2H), 7.55 (d, *J* = 8.3 Hz, 2H), 7.34 (d, *J* = 5.2 Hz, 1H), 6.81 (s, 1H), 5.65 (s, 2H), 5.26 (s, 2H), 3.60 (t, *J* = 8.1 Hz, 2H), 0.83 (t, *J* = 8.0 Hz, 2H), -0.11 (s, 9H); ^{13}C NMR (100 MHz, DMSO-*d*$_6$) δ: 162.0, 149.9, 143.7, 142.0, 136.5, 134.0, 130.7, 129.0 (2C), 128.5 (2C), 118.8, 117.0, 98.7, 70.8, 65.9, 64.4, 17.3, -1.5 (3C); IR (neat, cm^{-1}): 2950 (w), 2983 (w, br), 1724 (s, sh), 1557 (m), 1368 (m),

1248 (m), 1156 (s, br), 1075 (s, br), 856 (s), 833 (s); HRMS (APCI/ASAP, *m/z*): found 417.1398, calcd. for $C_{21}H_{26}N_2O_3SiCl$, $[M+H]^+$, 417.1401.

2,4-Bis(4-methoxyphenyl)-1-((2-(trimethylsilyl)ethoxy)methyl)-1*H*-pyrrolo[2,3-*b*]pyridine (15b)

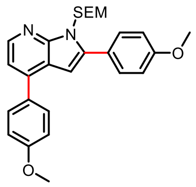

To a mixture of 4-chloro-2-iodo-1-((2-(trimethylsilyl)-ethoxy)methyl)-1*H*-pyrrolo[2,3-*b*]pyridine (**7**) (108 mg, 0.264 mmol), 4-methoxyphenylboronic acid (95 mg, 0.63 mmol), XPhos (7.8 mg, 0.016 mmol), XPhos Pd G2 pre-catalyst (13 mg, 0.016 mmol), K2CO3 (136 mg, 0.985 mmol) was added degassed 1,4-dioxane:water (1:1, 2.4 mL) under an N_2 atmosphere. The reaction mixture was stirred at 100 °C for 3 h before allowed to cool to rt. The solvent was removed in vacuo, and the residue was added EtOAc (10 mL) and water (10 mL). The layers were separated, and the aqueous layer was extracted with more EtOAc (3 × 10 mL). The combined organic phases were washed with brine (10 mL), dried over anhydrous Na_2SO_4, filtered and concentrated in vacuo. The product was purified by silica-gel column chromatography (CH_2Cl_2/MeOH, 99:1, R_f = 0.13) giving an orange oil, 100 mg (0.217 mmol, 82%); ^{1}H NMR (400 MHz, $CDCl_3$) δ 8.34 (d, J = 5.0 Hz, 1H), 7.73-7.70 (m, 4H), 7.15 (d, J = 5.0 Hz, 1H), 7.04-6.98 (m, 4H), 6.74 (s, 1H), 5.69 (s, 2H), 3.85 (s, 3H), 3.84 (s, 3H), 3.79 (t, J = 8.2 Hz, 2H), 0.99 (t, J = 8.2 Hz, 2H), -0.02 (s, 9H); ^{13}C NMR (100 MHz, $CDCl_3$) δ 159.8 (2C), 150.4, 143.0, 142.2, 140.7, 131.3, 130.6 (2C), 129.7 (2C), 124.6, 118.5, 115.6, 114.2 (2C), 114.1 (2C), 99.5, 70.8, 66.4, 55.3 (2C), 18.1, -1.4 (3C); IR (neat, cm^{-1}): 2951 (w), 2836 (w), 1609 (m), 1490 (m), 1245 (s), 1032 (m), 833 (s); HRMS (APCI/ASAP, m/z): found 461.2257, calcd. for $C_{27}H_{33}N_2O_3Si$, $[M+H]^+$, 461.2260.

((1-((2-(Trimethylsilyl)ethoxy)methyl)-1*H*-pyrrolo[2,3-b]pyridine-2,4-diyl)bis(4,1-phenylene))dimethanol (15c)

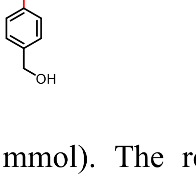

The reaction was performed as described for **15b** starting with 4-chloro-2-iodo-1-((2-(trimethylsilyl)-ethoxy)methyl)-1*H*-pyrrolo[2,3-*b*]pyridine (**7**) (277 mg, 0.678 mmol), (4-(hydroxymethyl)phenyl)boronic acid (227 mg, 1.50 mmol), XPhos (24.0 mg, 0.036 mmol), XPhos Pd G2 pre-catalyst (24 mg, 0.030 mmol). The reaction was run for 19 min at 90 °C. Purification by silica-gel column chromatography (CH_2Cl_2/MeOH, 95:5, R_f= 0.45) gave 246 mg (0.534 mmol, 79%) of an off-white solid; mp. 142.5-144 °C; ^{1}H NMR (400 MHz, DMSO-d_6) δ 8.36 (d, J = 5.0 Hz, 1H),

7.81-7.78 (m, 4H), 7.51 (d, J = 8.2 Hz, 2H), 7.46 (d, J = 8.2 Hz, 2H), 7.32 (d, J = 5.0 Hz, 1H), 6.86 (s, 1H), 5.68 (s, 2H), 5.29 (t, J = 5.7 Hz, 2H), 4.59 (t, J = 6.0 Hz, 4H), 3.67 (t, J = 8.1 Hz, 2H), 0.88 (t, J = 8.1 Hz, 2H), -0.07 (s, 9H); ^{13}C NMR (100 MHz, DMSO-d_6) δ 150.1, 143.2, 143.1 (2C), 141.9, 140.3, 136.2, 129.8, 128.6 (2C), 128.1 (2C), 127.1 (2C), 126.7 (2C), 117.5, 115.7, 99.7, 70.5, 65.8, 62.6, 62.5, 17.4, -1.4 (3C); IR (neat, cm^{-1}): 3404 (w, br), 2919 (w), 2858 (w), 1730 (w), 1585 (m), 1364 (m), 1245 (m), 1082 (s), 833 (s); HRMS (APCI/ASAP, *m/z*): found 461.2259, calcd. for $C_{27}H_{33}N_2O_3Si$, $[M+H]^+$, 461.2260.

3. Biochemical assays

CSF1R enzymatic inhibitory assay

The compounds were supplied in a 10 mM DMSO solution, and enzymatic CSF1R inhibition potency was determined by Invitrogen (ThermoFisher) using their Z'-LYTE® assay technology.[11] The assay is based on fluorescence resonance energy transfer (FRET). In the primary reaction, the kinase transfers the gamma-phosphate of ATP to a single tyrosine residue in a synthetic FRET-peptide. In the secondary reaction, a site-specific protease recognizes and cleaves non-phosphorylated FRET-peptides. Thus, phosphorylation of FRET-peptides suppresses cleavage by the development reagent. Cleavage disrupts FRET between the donor (i.e. coumarin) and acceptor (i.e., fluorescein) fluorophores on the FRET-peptide, whereas uncleaved, phosphorylated FRET-peptides maintain FRET. A ratiometric method, which calculates the ratio (the emission ratio) of donor emission to acceptor emission after excitation of the donor fluorophore at 400 nm, is used to quantitate inhibition. All compounds were first tested for their inhibitory activity at 500 nM in duplicates. The potency observed at 500 nM was used to set starting point of the IC_{50} titration curve, in which two levels were used 1000 or 10000 nM. The IC_{50} values reported are based on the average of at least 2 titration curves (minimum 20 data points), and were calculated from activity data with a four parameter logistic model using SigmaPlot (Windows Version 12.0 from Systat Software, Inc.) Unless stated otherwise the ATP concentration used was equal to K_M (ca 10 mM).

CSF1R enzymatic inhibitory assay (LANCE)

The TR-FRET-based LANCE *Ultra* assay (Perkin Elmer) was used to determine IC_{50} values for various CSF1R inhibitors. Kinase activity and inhibition in this assay was measured as

recommended by the manufacturer. Briefly, a specific Ultra ULight GT peptide substrate (50 nM final concentration) was allowed to get phosphorylated by CSF1R (0.5 nM final concentration) in enzymatic buffer (50 mM Hepes pH 7.5, 10 mM $MgCl_2$, 1 mM EGTA, 0.01% Tween 20, 2 mM dithiothreitol, 1% DMSO) containing ATP at 25 µM or 2.5 mM of the kinase for 1 h at room temperature. All compounds were tested in an 8-point dose response curve up to a final concentration of 10 µM. The compound transfer was facilitated via acoustic dispensing with the Echo 520 (Beckman Labcyte) using the Echo Dose Response software package. Subsequently, phosphorylation or inhibition was detected by addition of specific europium (Eu)-labelled anti-phospho-antibodies (2 nM), which upon binding to the phospho-peptide give rise to a FRET signal. The FRET signal was recorded in a time-resolved manner in a Perkin Elmer EnVision reader. All assays were performed in a final volume of 20 µl in low volume white 384 well plates from Corning (4513). All assay data was analysed with the Quattro Workflow software package from Quattro Research.

4. NMR spectra

NMR spectrum of 3a

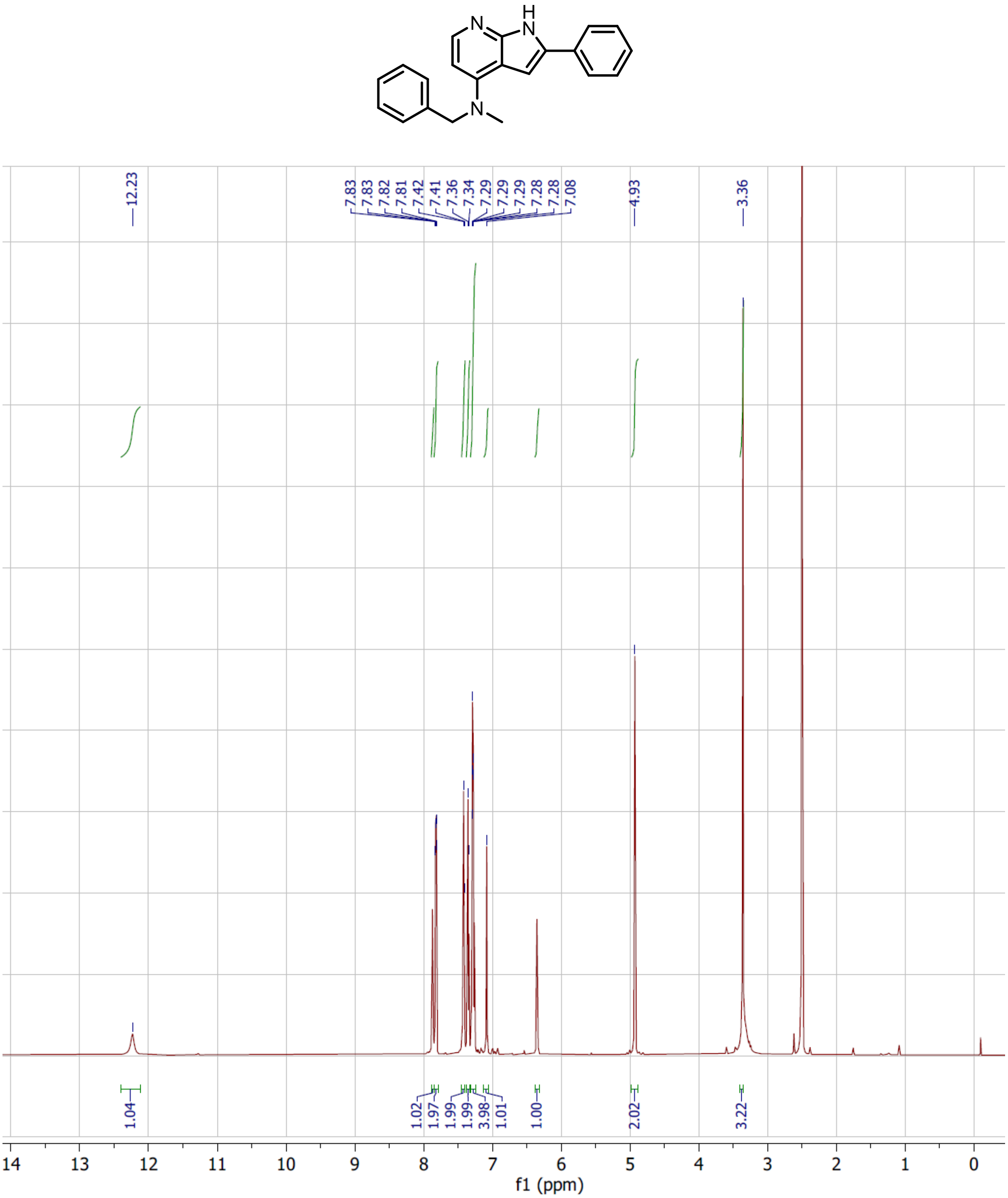

Figure S1. ^{1}H NMR (600 MHz, DMSO-d_6) of **3a**.

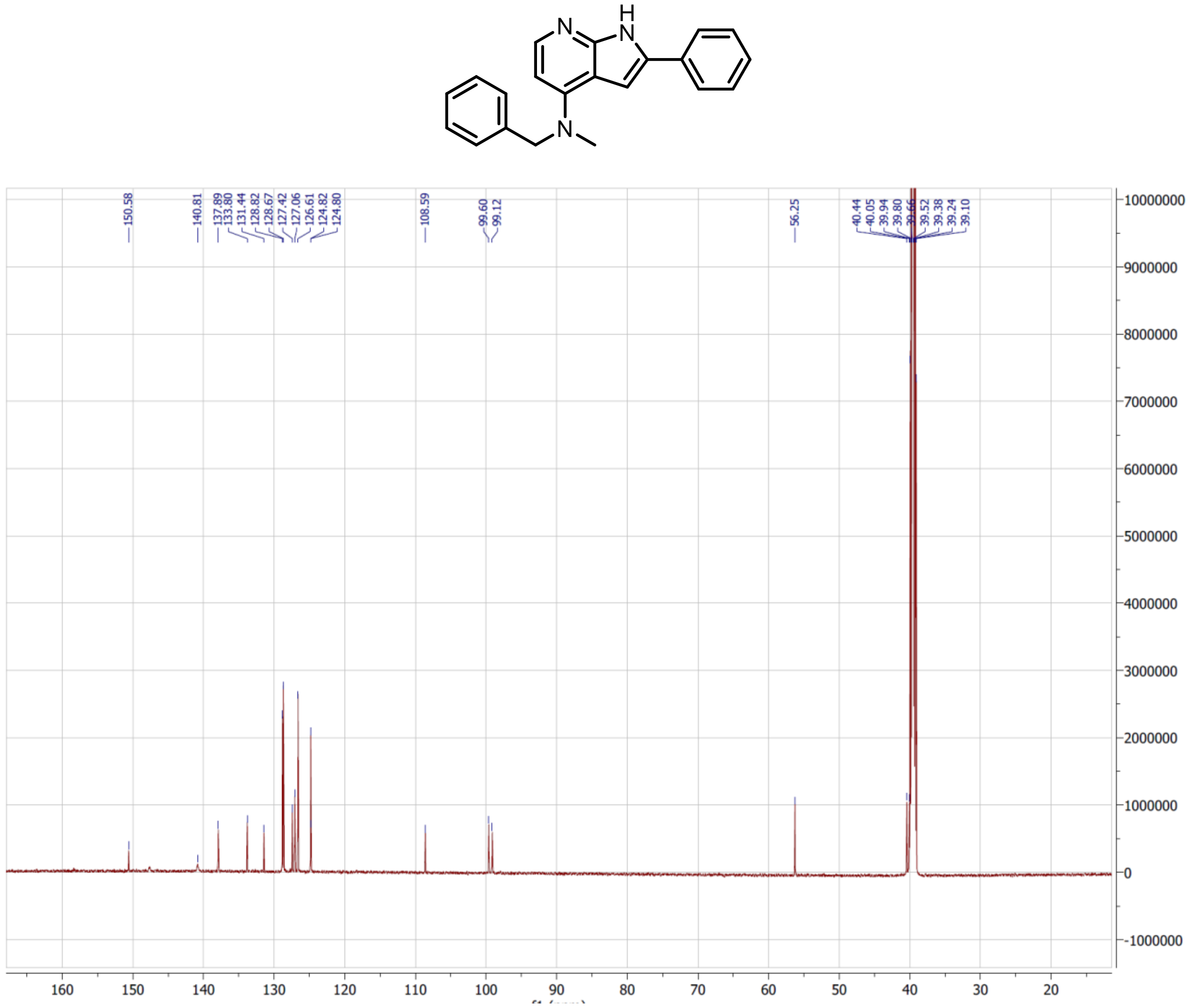

Figure S2. ^{13}C NMR (125 MHz, DMSO-d_6) of **3a**.

NMR data in $CDCl_3$:

^{1}H NMR (600 MHz, $CDCl_3$) δ 12.40 (s, br, 1H), 8.00 (d, J = 5.9 Hz,1H), 7.71 (d, J = 7.6 Hz, 2H), 7.40-7.35 (m, 4H), 7.32-7.25 (m, 4H), 6.75 (s, 1H), 6.27 (d, J = 5.9 Hz, 1H), 4.85 (s, 2H), 3.24 (s, 3H); ^{13}C NMR (150 MHz, $CDCl_3$) δ 151.0 (2C), 143.6, 137.8, 135.0, 132.4, 128.8 (4C), 127.4, 127.3, 126.8 (2C), 125.4 (2C), 109.8, 99.8, 98.1, 57.1, 39.4.

NMR spectrum of 3b

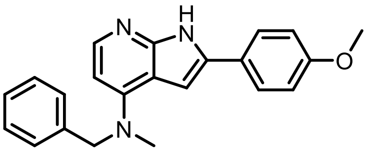

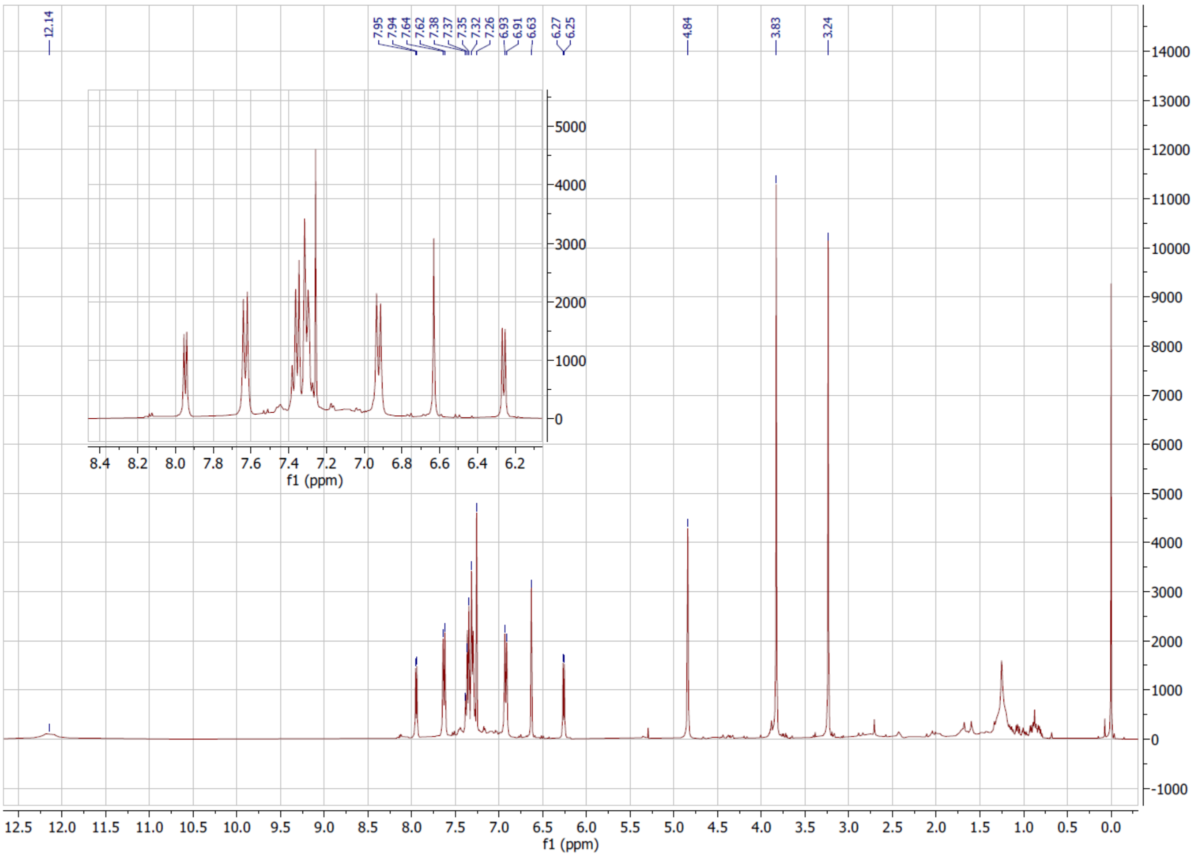

Figure S3. ^{1}H NMR (400 MHz, $CDCl_3$) of **3b**. The compound contains some aliphatic impurities.

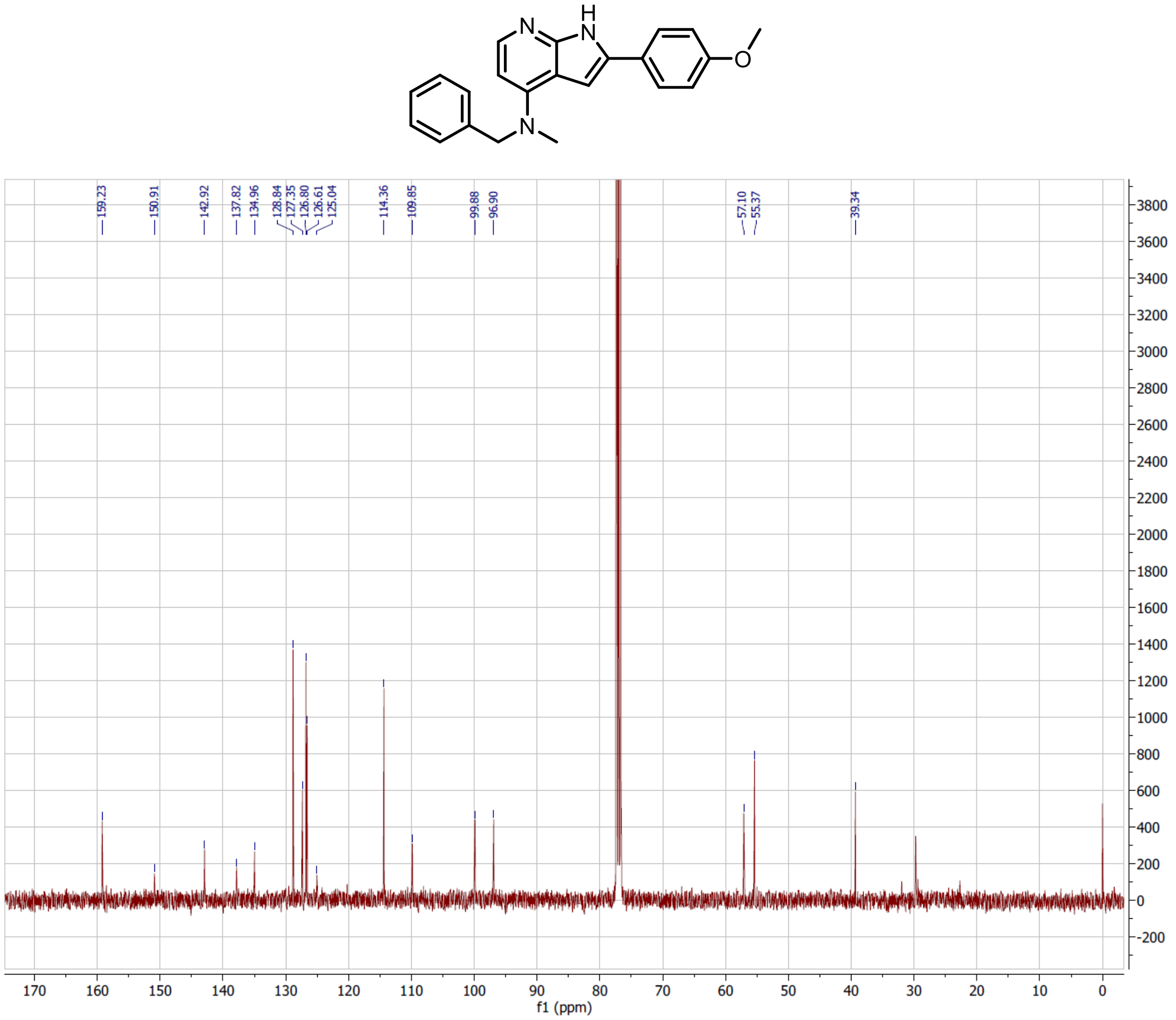

Figure S4. ^{13}C NMR (100 MHz, $CDCl_3$) of **3b**. The compound contains some aliphatic impurities.

NMR spectrum of 3c

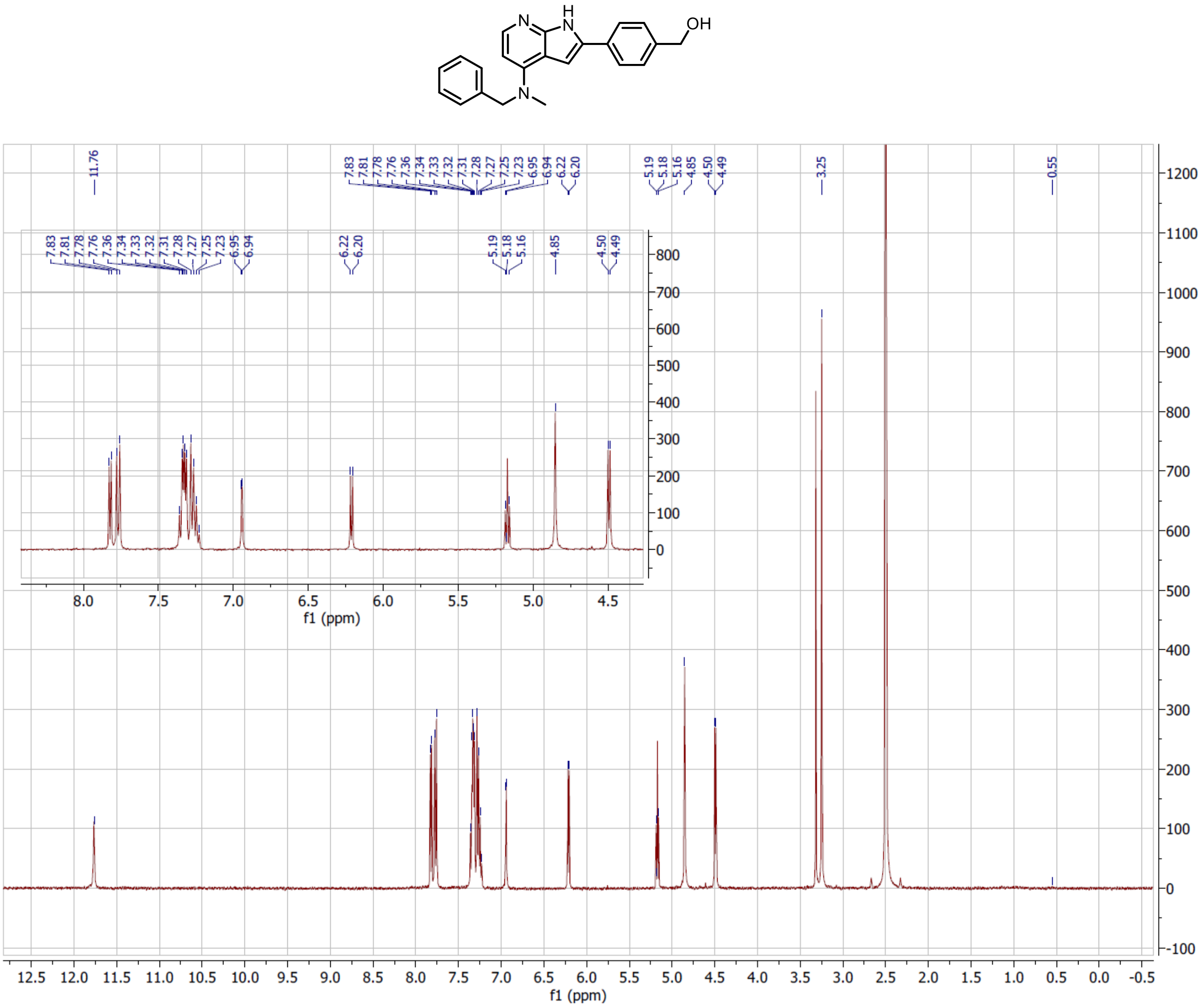

Figure S5. ^{1}H NMR (DMSO-d6, 400 MHz) of **3**.

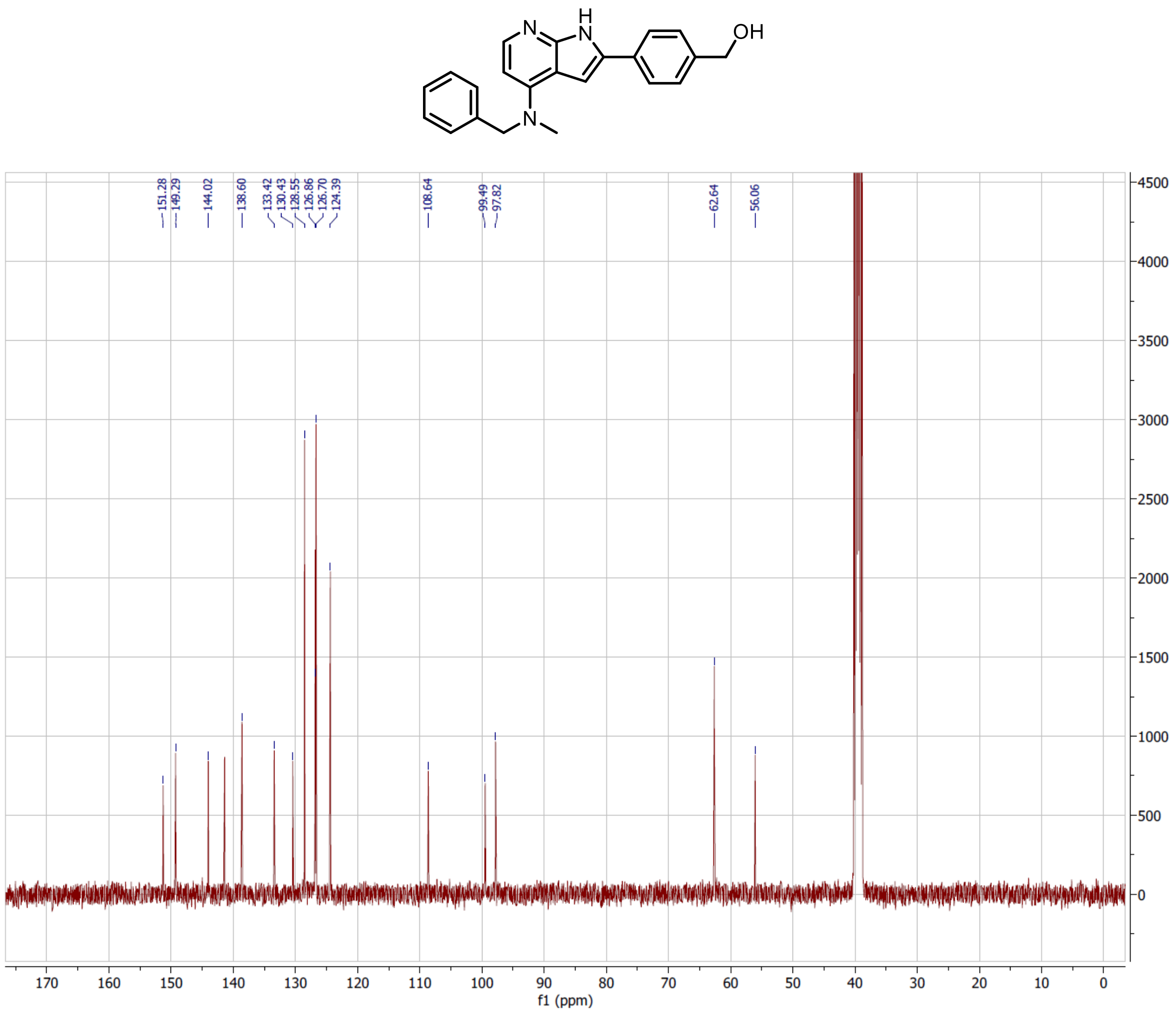

Figure S6. ^{13}C NMR (100 MHz DMSO-d_6) of **3c**.

NMR spectra of 16a

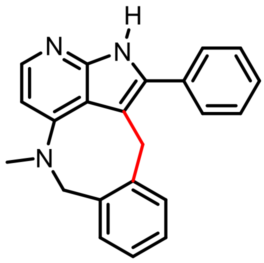

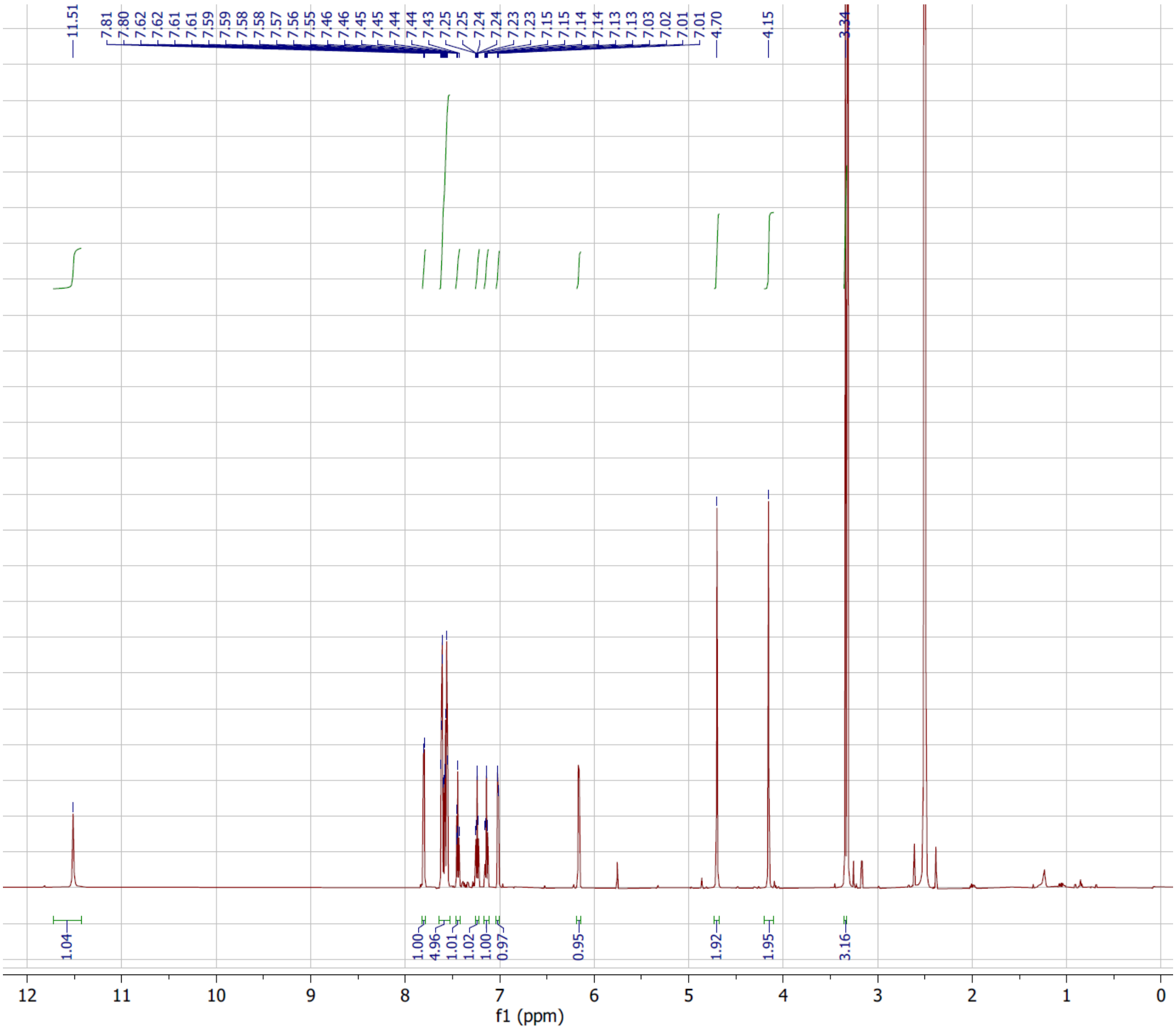

Figure S7. ^{1}H NMR (DMSO-d_6, 600 MHz) of **16a**.

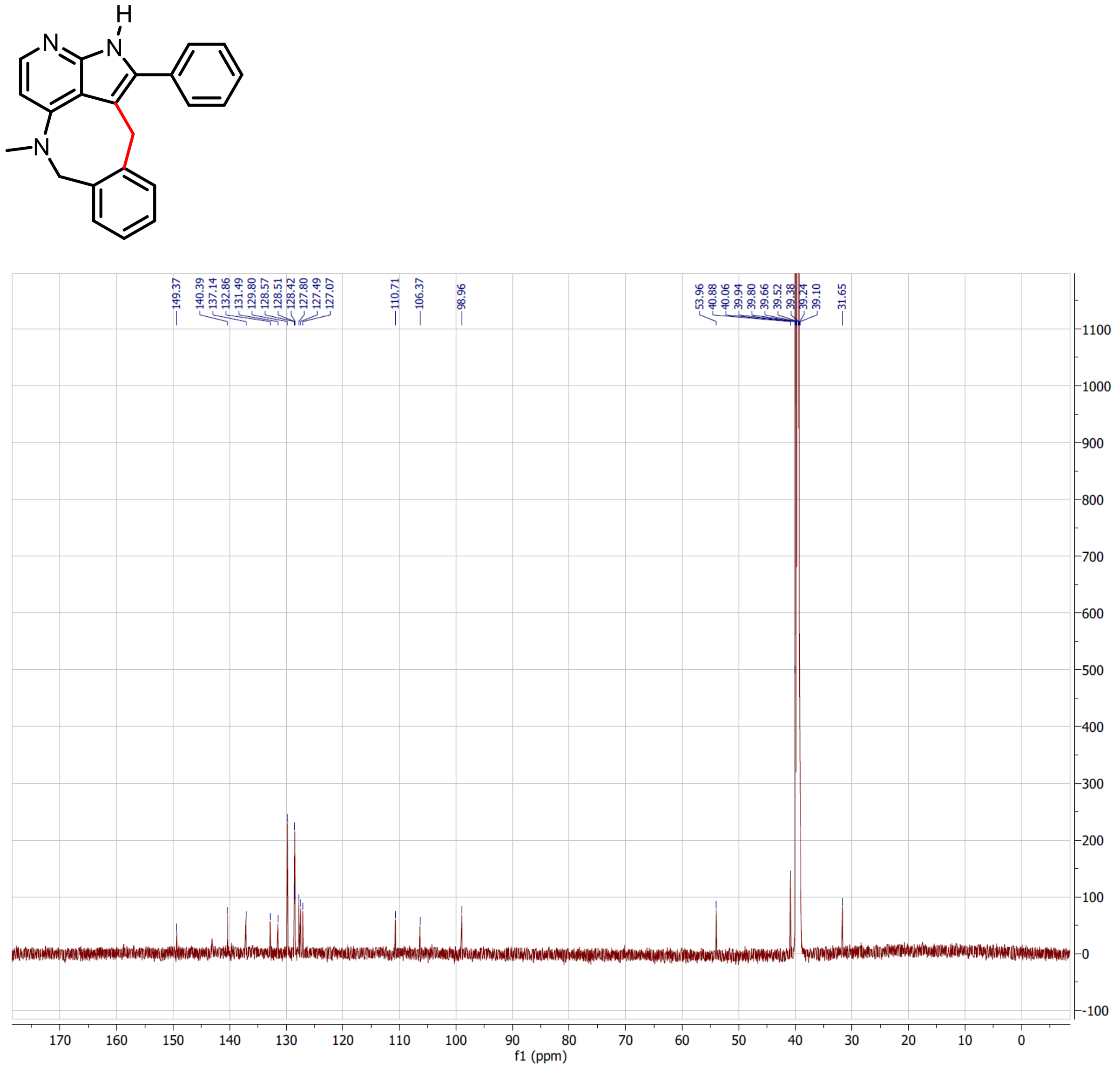

Figure S8. ^{13}C NMR (125 MHz DMSO-d_6) of **16a**.

NMR spetra of 16c

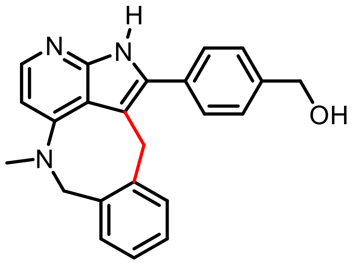

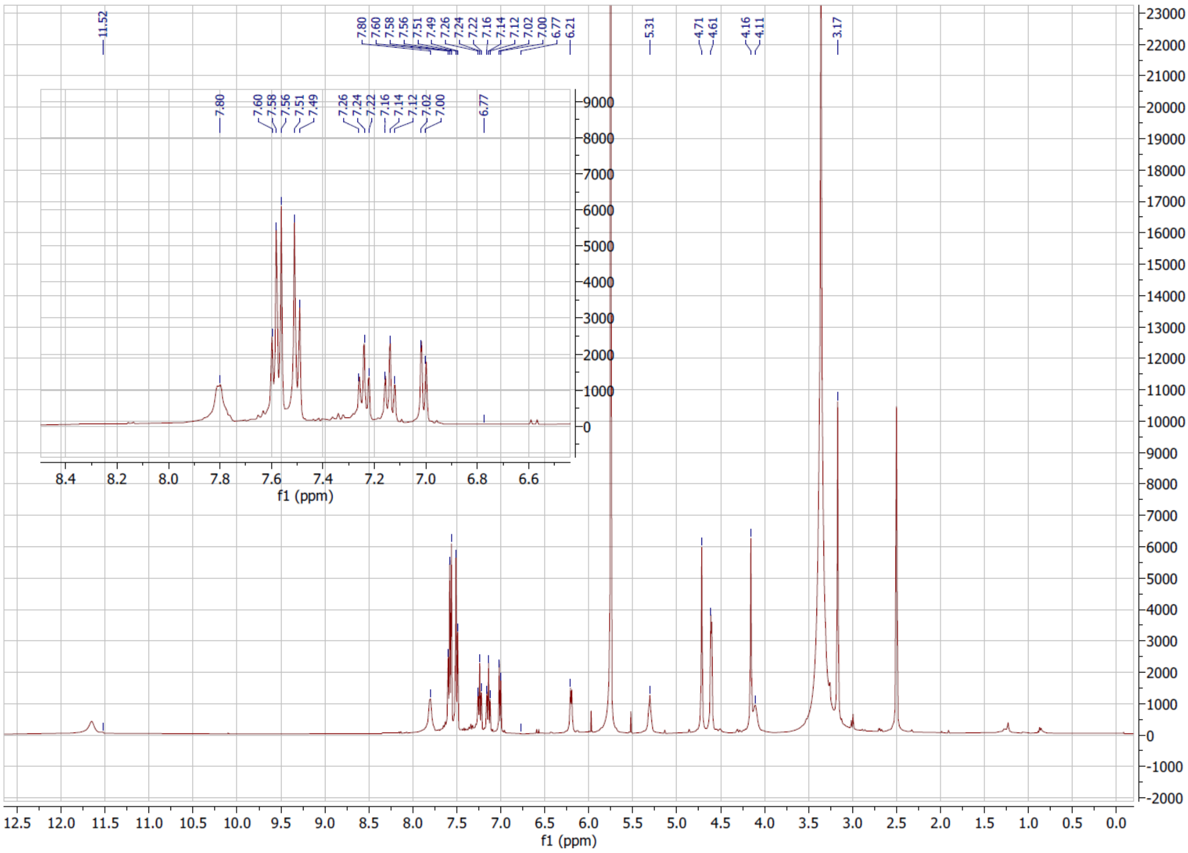

Figure S9. ^{1}H NMR (400 MHz DMSO-d$_6$) of **16c**. The material contains residual CH_2Cl_2 (5.76 ppm) from purification.

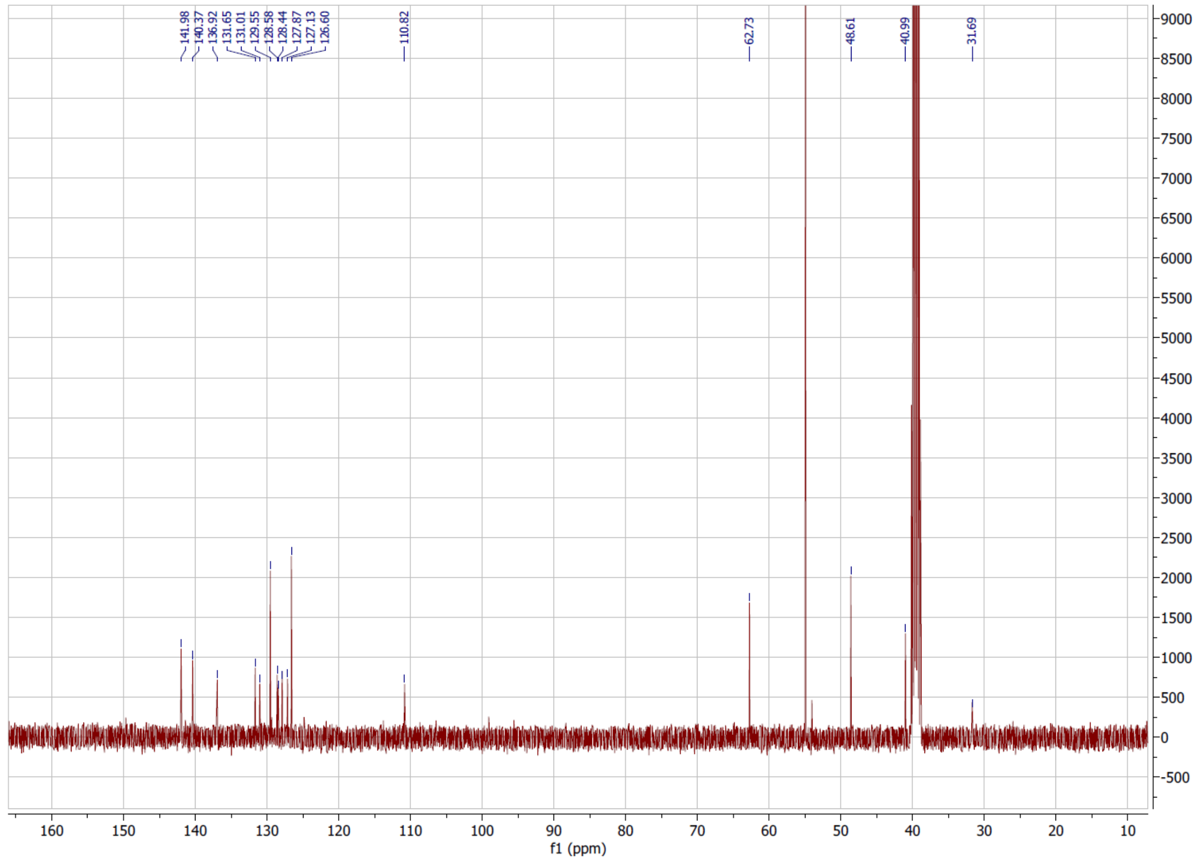

Figure S10. ^{13}C NMR (100 MHz DMSO-d_6) of **17c**. The material contains residual CH_2Cl_2 (54 ppm) from purification.

Structural assignment of compound 16c.

The structure of compound **6c** with numbered positions is given in Figure S11.

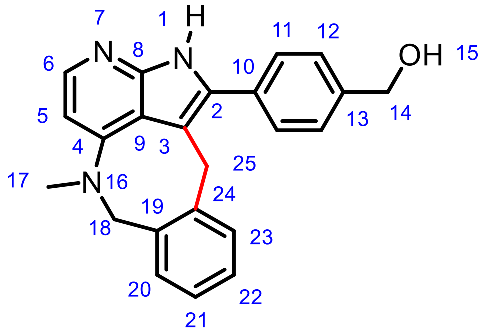

Figure S11. The suggested structure of compound **16c** with atom numbering.

As compound **16c** contained a rare eight-membered ring, the details of the structural elucidation of this structure will therefore be presented.

Firstly, the broad singlet with integral 1 at 11.65 ppm was assigned to the H-1 amine proton. A signal at 5.30 ppm with integral 1 showed no coupling in the HSQC spectrum, and was therefore assigned to the hydroxyl group H-15. This hydroxyl signal showed coupling in the COSY spectrum to a doublet at 4.61 ppm (J = 4.2 Hz, 2H) which was assigned to H-14, and the corresponding C-14 signal was found at 62.7 ppm. This benzylic CH_2-group showed ^{1}H-^{13}C long range coupling in the HMBC spectrum to a substituted sp^2-carbon at 142.0 ppm, which was assigned to C-13. Long range coupling was also seen between H-14 and a carbon signal at 126.6 ppm. This signal corresponded to the doublet signal with integral 2 at 7.50 ppm (J = 8.1 Hz), and was assigned to H-12. The COSY spectrum revealed coupling between H-12 and a multiplet signal with integral 3 at 7.60-7.56 ppm. This multiplet contained two doublet signals overlapping, and one of these doublets was assigned to H-11. The corresponding C-11 signal was found at 129.5 ppm. ^{1}H-^{13}C long range coupling was observed between H-12 and 131.0 ppm, a substituted sp^2-carbon which was assigned to C-10. An aromatic carbon lacking hydrogen at 131.6 ppm showed long range coupling to H-11, and was assigned to C-2.

A broad doublet at 7.81 ppm with integral 1 was assigned to H-6 because of the high chemical shift, and because the signal at H-6 was typically observed in this region. The corresponding C-6 signal was found from the HSQC spectrum at 142.1 ppm, though this signal was too weak to be observed in the ^{13}C NMR spectrum. Weak long-range coupling was seen between H-6 and a carbon signal at 150.3 ppm, which was assigned to the C-8. The H-6 peak showed ^{1}H-^{1}H coupling in the COSY spectrum to a doublet at 6.20 ppm (J = 5.8 Hz). This signal was in turn assigned to H-5, and the corresponding C-5 was found at 99.0 ppm. H-5 showed ^{1}H-^{13}C long-range coupling to two carbon signals at 41.0 and 106.4 ppm. The signal at 106.4 ppm was assigned to C-9.

The HSQC spectrum revealed that the carbon signal at 41.0 ppm corresponded to a proton signal overlapping with the water residue signal in the proton spectra, at 3.25 ppm. The chemical shifts of these signals indicated that they belong to the methyl group in position 17. Long range coupling was seen between H-17 and the carbon signals at 149.7 and 54.0 ppm. The signal at 149.7 ppm was assigned to C-4, and the signal at 48.6 ppm was assigned to the benzylic C-18. The HSQC spectrum showed that the two-proton singlet at 4.71 ppm belonged to H-18.

The C-9 signal showed long-range coupling to a singlet at 4.16 ppm containing two protons. Such a coupling in this region had not been observed in any of the spectra of the other compounds. The corresponding carbon was found from the HSQC spectrum at 31.7 ppm. This peak showed coupling to C-9, C-2 and a carbon at 110.9. The signal at 110.9 ppm was assigned to C-3. In the other compounds described, the 3-position had contained a proton signal between 6.7-7.0 ppm. In compound **XX**, the atom C-3 signal is substituted. The mentioned signals at 4.16 ppm/31.7 ppm were therefore assumed to be connected in this position, and were assigned to position 25.

The CH_2-group at position 25 showed long-range coupling to two substituted carbons at 136.9 and 140.4 ppm, which also had coupling to H-18. The CH_2-group was therefore clearly connected to the pyrrole-unit in position 3, and the aromatic part of the 4-amino group. The splitting and coupling pattern of the remaining signals in the aromatic region indicated that an *ortho*-substitution had taken place in this phenyl group. This confirmed the suspicion of an eight-membered-ring formation. A part of the HMBC spectrum is shown in Figure S12, depicting the long-range couplings observed for the bridging CH_2 at H-25.

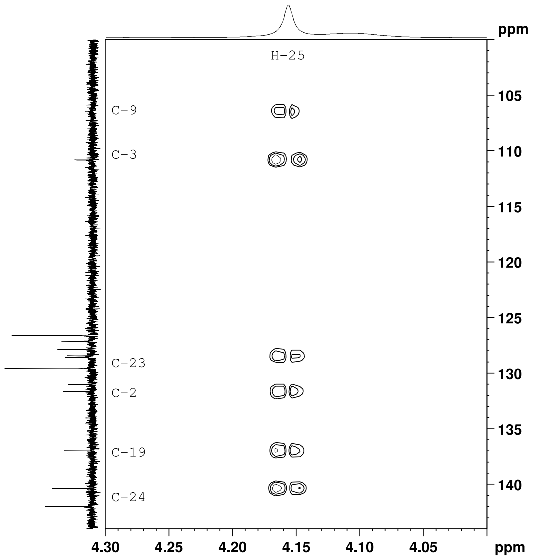

Figure S12 Part of the HMBC spectrum showing the ^{1}H-^{13}C long range couplings of the singlet at 4.16 ppm, assigned to position 25.

Remaining signals in the aromatic region were at this point two triplets at 7.24 ppm (J = 7.4 Hz) and 7.14 ppm (J = 7.3 Hz), one doublet at 7.00 ppm (J = 7.3 Hz), as well as the remaining doublet overlapping in the multiplet at 7.60-7.56 ppm (1H).

Long range coupling was detected between C-18 and the multiplet at 7.60-7.56 ppm (overlap with H-11), which was assigned to H-20. The corresponding C-20 signal was found at 128.6 ppm. Long range coupling was also observed between the bridge methylene C-25 and the doublet at 7.00 ppm (J = 7.3 Hz), which was assigned to H-23. The corresponding carbon signal was found at 128.4 ppm. From ^{1}H-^{1}H couplings in the COSY spectrum, the signals at 7.24 ppm/127.1 ppm and 7.14 ppm/127.9 ppm were assigned to positions 21 and 22, respectively. Long range coupling between all *meta*-positions of the phenyl ring was observed in the HMBC spectrum. This observation provided the basis for assigning the signal at 140.4 ppm to C-24, and 136.9 ppm to C-19. A part of the HMBC spectrum showing long range couplings in the aromatic region is shown in Figure S13. A summary of couplings that helped identify the structure is shown in Figure S14.

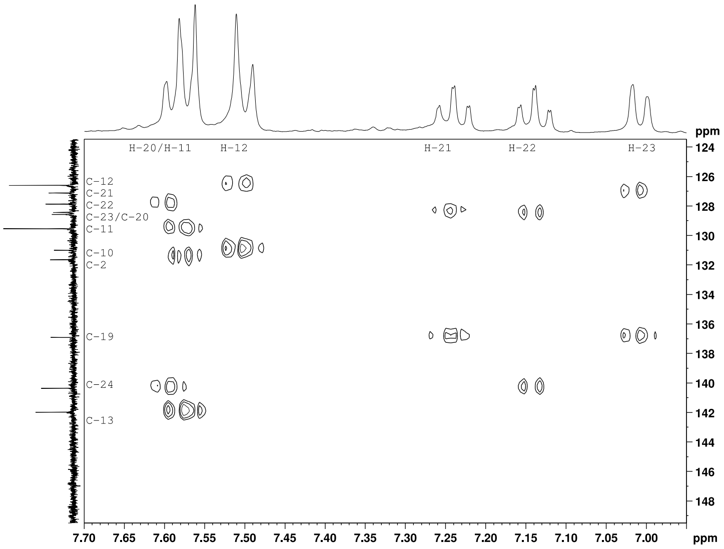

Figure S13. Part of the HMBC spectrum showing ^{1}H-^{13}C long range couplings of the aromatic region.

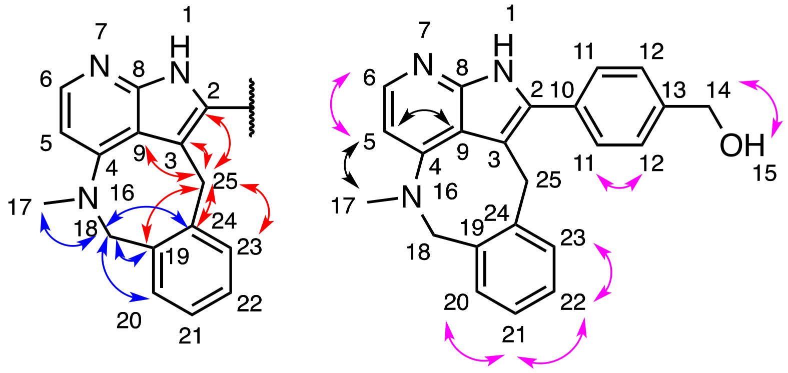

Figure S14. Left: The ^{1}H-^{13}C long range coupling observed in the HMBC spectra for H-18 (blue arrows) and H-25 (red arrows). Right: ^{1}H-^{1}H couplings observed in the COSY spectrum (pink arrows), and ^{1}H-^{13}C long range coupling of H-5 (black arrows).

All assigned ^{1}H and ^{13}C NMR shifts of compound **16c** are given in Table S**1**.

Table S1: ^{1}H and ^{13}C NMR for compound **16** (DMSO-d_6, 400 MHz for ^{1}H).

Position	^{1}H [ppm]	^{13}C [ppm]	COSY	HMBC
**1**	11.65 (s, 1H)	-		-
**2**	-	131.6		11, 25
**3**	-	110.8		25
**4**	-	149.7[a]		17, 18
**5**	6.20 (d, J = 5.8 Hz, 1H)	99.0	5	9, 17
**6**	7.81 (m, 1H)	142.1[a]	6	-
**8**	-	150.3[a]		-
**9**	-	106.4		5, 25
**10**	-	131.0		12
**11**	7.60-7.56 (m, 2H)[b]	129.5	12	2, 11, 13
**12**	7.50 (d, J = 8.1 Hz, 2H)	126.6	11, 14	10, 12, 14
**13**	-	142.0		11, 14
**14**	4.61 (d, J = 4.2 Hz, 2H)	62.7	12, 15	12, 13
**15**	5.30 (m, 1H)	-	14	-
**17**	3.25 (s, 3H)[c]	41.0		4, 5, 18
**18**	4.71 (s, 2H)	48.6		4, 17, 19, 20, 24
**19**	-	136.9		18, 21, 23, 25
**20**	7.60-7.56 (m, 1H)[b]	128.6	21	18, 22, 24
**21**	7.24 (t, J = 7.4 Hz, 1H)	127.1	20, 22	19, 23
**22**	7.14 (t, J = 7.3 Hz, 1H)	127.9	21, 23	20, 24
**23**	7.00 (d, J = 7.3 Hz, 1H)	128.4	22	19, 21, 25
**24**	-	140.4		18, 20, 22, 25
**25**	4.16 (s, 2H)	31.7		2, 3, 9, 19, 23, 24

[a]Not visible in ^{13}C NMR spectrum
[b]Overlapping signals
[c]Overlapping with water residue signal

Compound 17c

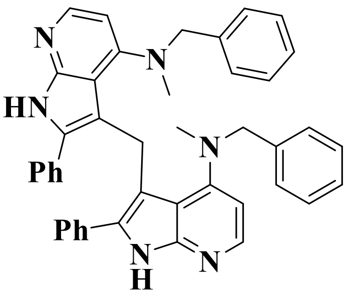

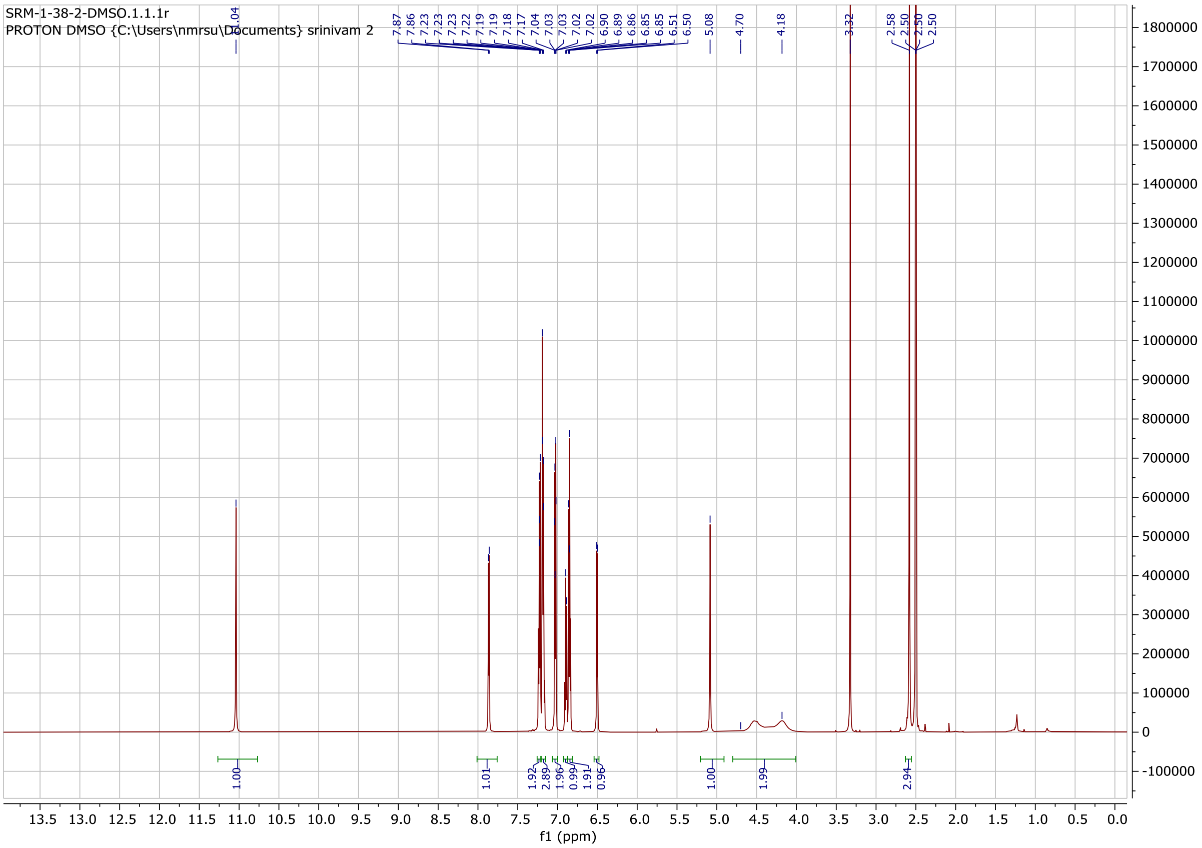

Figure S14. ^{1}H NMR (600 MHz DMSO-d_6) of **17c**.

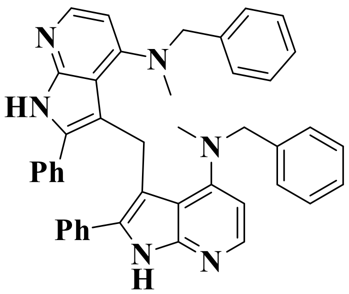

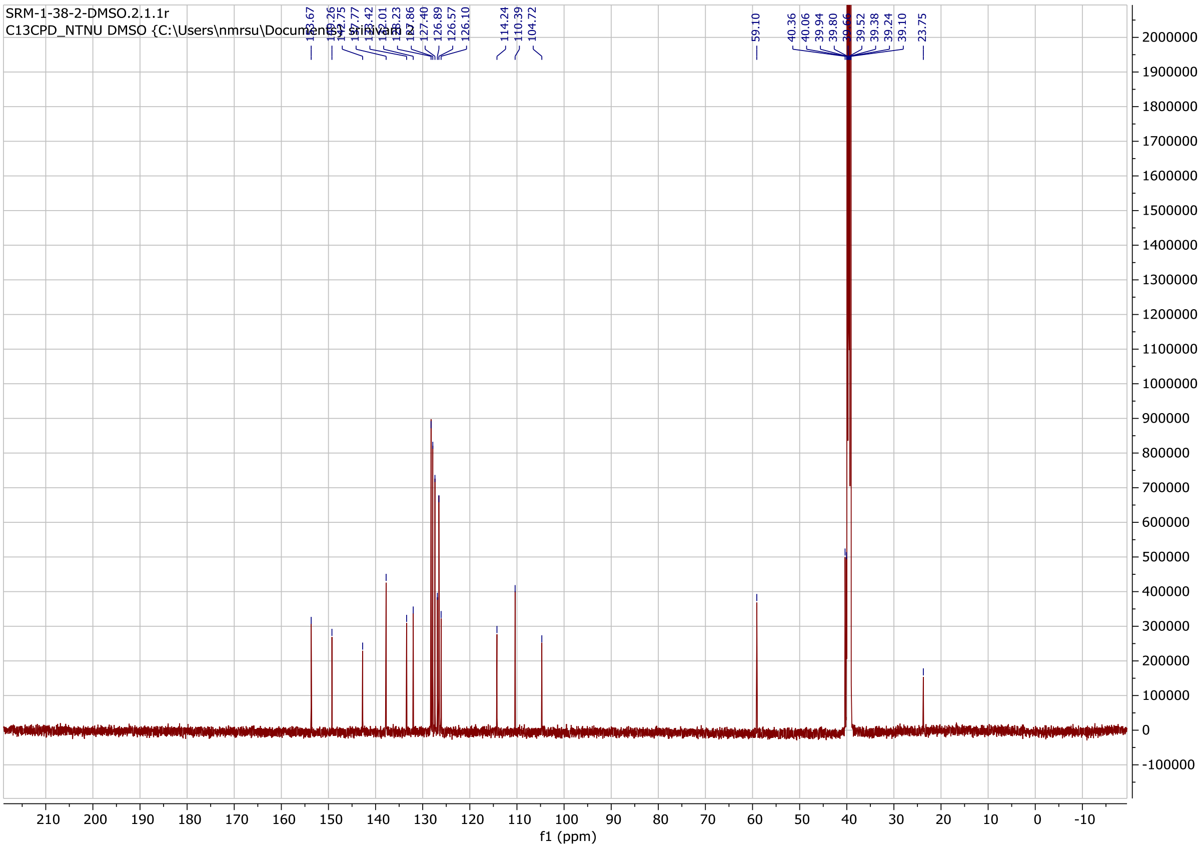

Figure S15. ^{13}C NMR (150 MHz DMSO-d$_6$) of **17c**.

5. References

[1] X. Wang, B. Zhi, J. Baum, Y. Chen, R. Crockett, L. Huang, S. Eisenberg, J. Ng, R. Larsen, M. Martinelli, P. Reider, A Practical Synthesis of 2-((1H-Pyrrolo[2,3-b]pyridine-4-yl)methylamino)-5-fluoronicotinic Acid, J. Org. Chem., 2006, **71**, 4021-4023, https://doi.org/10.1021/jo0602571.
[2] M. Layek, V. Gajare, D. Kalita, A. Islam, K. Mukkanti, M. Pal, A highly effective synthesis of 2-alkynyl-7-azaindoles: Pd/C-mediated alkynylation of heteroaryl halides in water, Tetrahedron, 2009, **65**, 4814-4819, https://doi.org/10.1016/j.tet.2009.04.054.
[3] M. Juchum, M. Guenther, E. Doering, A. Sievers-Engler, M. Laemmerhofer, S. Laufer, Trisubstituted Imidazoles with a Rigidized Hinge Binding Motif Act As Single Digit nM Inhibitors of Clinically Relevant EGFR L858R/T790M and L858R/T790M/C797S Mutants: An Example of Target Hopping, J. Med. Chem., 2017, **60**, 4636-4656, https://doi.org/10.1021/acs.jmedchem.7b00178.
[4] D. Yang, H.B. Jeon, Convenient N-formylation of amines in dimethylformamide with methyl benzoate under microwave irradiation, Bull. Korean Chem. Soc., 2010, **31**, 1424-1426, https://doi.org/10.5012/bkcs.2010.31.5.1424.
[5] C. Barberis, N. Moorcroft, C. Arendt, M. Levit, S. Moreno-Mazza, J. Batchelor, I. Mechin, T. Majid, Discovery of N-substituted 7-azaindoles as PIM1 kinase inhibitors - Part I, Bioorg. Med. Chem. Lett., 2017, **27**, 4730-4734, https://doi.org/10.1016/j.bmcl.2017.08.069.
[6] M.I. El-Gamal, M.S. Abdel-Maksoud, M.M.G. El-Din, K.H. Yoo, D. Baek, C.-H. Oh, Cell-Based Biological Evaluation of a New Bisamide FMS Kinase Inhibitor Possessing Pyrrolo[3,2-c]pyridine Scaffold, Arch. Pharm. , 2014, **347**, 635-641, https://doi.org/10.1002/ardp.201400051.
[7] C.-C. Cheng, C.-P. Chang, W.-S. Yu, F.-T. Hung, Y.-I. Liu, G.-R. Wu, P.-T. Chou, Comprehensive Studies on Dual Excitation Behavior of Double Proton versus Charge Transfer in 4-(N-Substituted amino)-1H-pyrrolo[2,3-b]pyridines, J. Phys. Chem. A, 2003, **107**, 1459-1471, https://doi.org/10.1021/jp021243b.
[8] Y. Nakajima, T. Tojo, M. Morita, K. Hatanaka, S. Shirakami, A. Tanaka, H. Sasaki, K. Nakai, K. Mukoyoshi, H. Hamaguchi, F. Takahashi, A. Moritomo, Y. Higashi, T. Inoue, Synthesis and evaluation of 1H-pyrrolo[2,3-b]pyridine derivatives as novel immunomodulators targeting Janus kinase 3, Chem. Pharm. Bull., 2015, **63**, 341-353, https://doi.org/10.1248/cpb.c15-00036.
[9] J.L. Henderson, S.M. McDermott, S.L. Buchwald, Palladium-Catalyzed Amination of Unprotected Halo-7-azaindoles, Org. Lett, 2010, **12**, 4438-4441, https://doi.org/10.1021/ol101928m.
[10] I.M. Bell, S.N. Gallicchio, M.R. Wood, A.G. Quigley, C.A. Stump, C.B. Zartman, J.F. Fay, C.-C. Li, J.J. Lynch, E.L. Moore, S.D. Mosser, T. Prueksaritanont, C.P. Regan, S. Roller, C.A. Salvatore, S.A. Kane, J.P. Vacca, H.G. Selnick, Discovery of MK-3207: A Highly Potent, Orally Bioavailable CGRP Receptor Antagonist, ACS Med. Chem. Lett., 2010, **1**, 24-29, https://doi.org/10.1021/ml900016y.
[11] B.A. Pollok, B.D. Hamman, S.M. Rodems, L.R. Makings, Optical probes and assays, WO 2000066766 A1, (2000).
